# Non-*aureus* staphylococci and mammaliicocci as a cause of mastitis in domestic ruminants: current knowledge, advances, biomedical applications, and future perspectives – a systematic review

**DOI:** 10.1007/s11259-023-10090-5

**Published:** 2023-03-25

**Authors:** Rocio Angélica Ruiz-Romero, Einar Vargas-Bello-Pérez

**Affiliations:** 1 Departamento de Medicina y Zootecnia de Rumiantes, Facultad de Medicina Veterinaria y Zootecnia, Universidad Nacional Autónoma de México, Av. Universidad 3000, Ciudad de México, 04510 México; 2 School of Agriculture, Policy and Development, University of Reading, New Agriculture Building, Earley Gate, Whiteknights Road, PO Box 237, Reading, Berkshire, RG6 6EU UK

**Keywords:** Mammary gland infection, Cows, Goats, Ewes, *Staphylococcus* spp, *Mammaliicoccus* spp, Biofilm prevention, Antimicrobial resistance

## Abstract

Non-*aureus* staphylococci and mammaliicocci (NASM) are one of the most common causes of subclinical mastitis in dairy animals and the extent of damage by intramammary infections (IMI) caused by NASM is still under debate. The different effects of NASM on the mammary gland may be associated with differences between bacterial species. NASM are normal and abundant colonizers of humans and animals and become pathogenic only in certain situations. The veterinary interest in NASM has been intense for the last 25 years, due to the strongly increasing rate of opportunistic infections. Therefore, the objective of this review is to provide a general background of the NASM as a cause of mastitis and the most recent advances that exist to prevent and fight the biofilm formation of this group of bacteria, introduce new biomedical applications that could be used in dairy herds to reduce the risk of chronic and recurrent infections, potentially responsible for economic losses due to reduced milk production and quality. Effective treatment of biofilm infection requires a dual approach through a combination of antibiofilm and antimicrobial agents. Even though research on the development of biofilms is mainly focused on human medicine, this technology must be developed at the same time in veterinary medicine, especially in the dairy industry where IMI are extremely common.

## Introduction

Non-*aureus* staphylococci and mammaliicocci (NASM) are part of a large group of Gram-positive bacteria that share their mutual lack of the coagulase virulence factor (El-Jakee et al. 2013; Michels et al. 2021; Pyörälä et al. 2009). Therefore, there are *Staphylococcus chromogenes* isolates that can clot plasma, since the main pathogen causing mastitis in cows is coagulase-positive *S. aureus*, the coagulase-positive phenotype of *S. chromogenes* can easily lead to misidentification (Dos Santos et al. 2016). Staphylococci are part of the normal skin flora of animals and have been isolated from different body sites from cows like hair coat, nares, teat skin, teat canal, and from the dairy environment such as bedding as well as on the milker´s hands (Taponen et al., 2009). Interest in this group of bacteria has increased over the last years both in humans and in veterinary medicine (Pyörälä et al. 2009). In recent years, this group has become one of the main pathogens causing mastitis in domestic ruminants (Dalanezi et al. 2020). The spread and prevalence of mastitis caused by NASM vary from country to country, due to season dynamics and weather changes that affect the proportion of NASM pathogens that are spread among herds (Naqvi et al. 2018). Furthermore, the type of housing system, parity, lactation stage, variation in sampling techniques, level of farm production intensity, and method of species identification are other factors that influence the spread and prevalence of mastitis caused by NASM (El-Jakee et al. 2013; Vanderhaeghen et al. 2014a).

For many years, this group has been managed as a minor group, however, some studies propose that infections with NASM may cause more harm to the mammary gland level than thought before. Therefore, there is a need to identify subgroups and species-specific at a herd level to improve our understanding of the differences between species and thus, improve therapeutic success (Condas et al. 2017; Pyörälä et al. 2009; Schukken et al., 2009). Research relying on genotypic identification demonstrated the existence of a vast NASM species in different ruminants, environment, milk samples, and udder-related habitats (Vanderhaeghen et al. 2015). Often, NASM occurs more in subclinical mastitis when compared to clinical mastitis. Lack of consciousness of farmers on the potential threat of NASM to cause mastitis infection may be the reason for mastitis problems, information on the prevalence of NASM and the ability to form biofilms (that contribute to more antimicrobial resistance) would be useful for the control of mastitis caused by NASM (Koop et al. 2012; Lee at al., 2022).

The objective of this review was to discuss the importance of the NASM in intramammary infections, the importance of making a correct identification, the advances that exist to prevent the formation of biofilms, as well as the different methodologies available to diagnose them and offer alternative treatments that eliminate the NASM found within biofilms. This will offer some insights on how to avoid the formation of multi-resistant bacteria to antimicrobials so that they can be applied in veterinary medicine.

## Materials and methods

### Search strategy and selection criteria

Our search for information focused on studies reporting mastitis in ruminants caused by NASM and advances in the prevention of biofilm formation. A database was created from studies specifying the following topics: non-*aureus* staphylococci and mammaliicocci as a cause of mastitis in domestic ruminants, phenotypic and genotypic identification, pathogenesis, virulence factors, antimicrobial resistance, biofilm formation, and biofilms treatment, covered the years 2000–2022, the search was conducted between July 2022 to October 2022.

The publications were obtained from databases such as PubMed, ScienceDirect, Scopus, Springer Link, Wiley Online Library, Scielo, Science Research, Redalyc, and Google Academic and we only considered full-length research articles and review papers. Obtaining information to find relevant publications was based on a chain of specific topics like mastitis. The search string with the topic was supported by Boolean operators (“AND”, “OR”), which served to specify the required information. All search terms within a string were checked for a “title, abstract, and keyword”. The keywords used were mastitis, cow, goat, sheep, ewe, doe, non-*aureus* staphylococci, mammaliicocci, phenotypic identification, genotypic identification, pathogenesis, virulence factors, antimicrobial resistance, biofilm formation, and biofilm treatment. No specific research articles on small ruminants related to the focus of the review were found, this is probably because the prevalence of subclinical mastitis in small ruminants averages from 5 to 30%, and the annual incidence of clinical mastitis is generally lower than 5% and according to some reports, the prevalence of subclinical mastitis in cows could be higher (> 61%) (Contreras et al. 2007; Cervinkova et al. 2013).

**Fig. 1:**
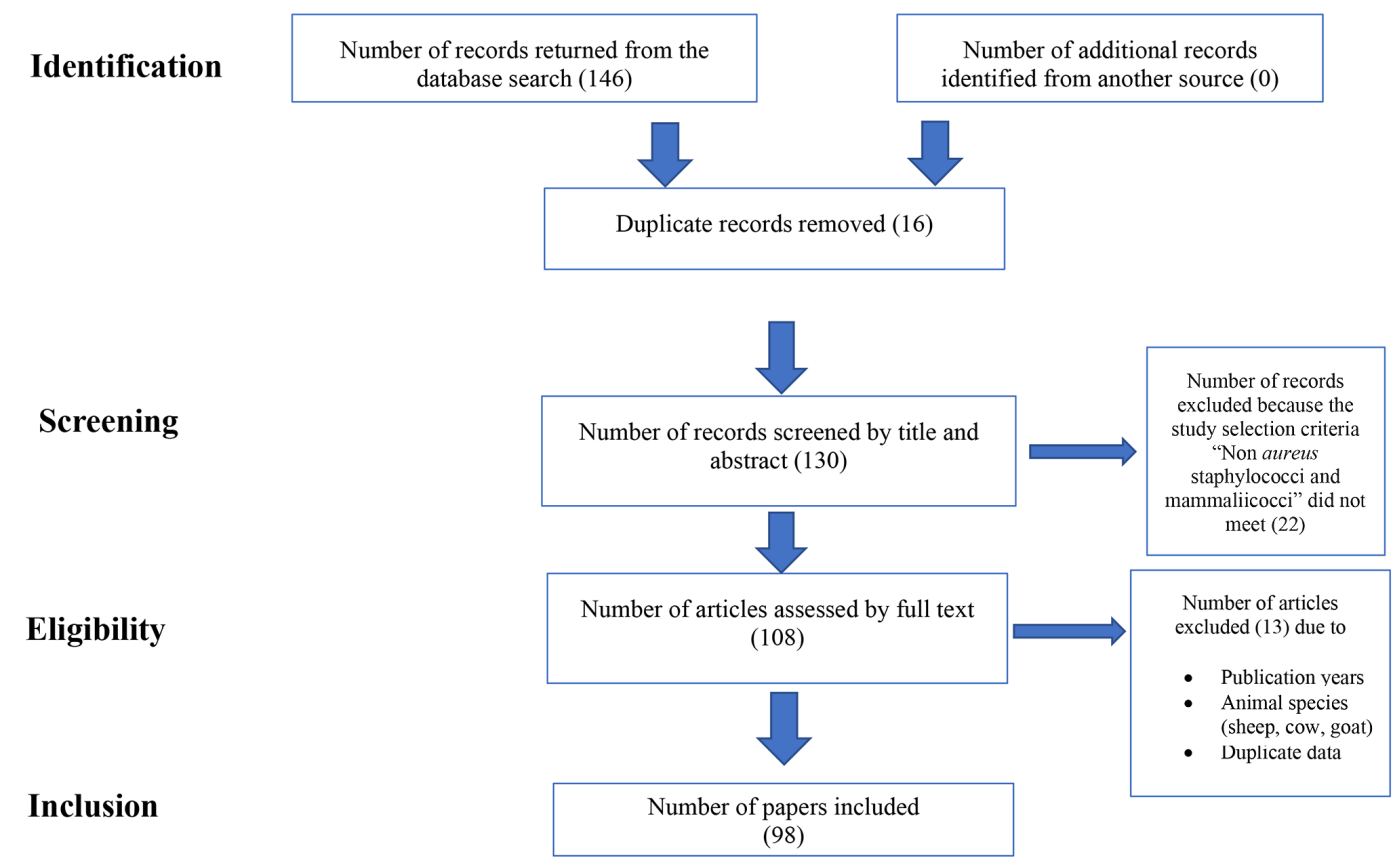
PRISMA study flow of the selection process of the literature from initial search and screening to final selection of publications to be included in the review

The publications that were eliminated because of duplicity were 16, and 22 publications were not considered in the present paper, because they did not have enough data or failed to report the final identification of the Non-*aureus* staphylococci and mammaliicocci, techniques used to identify the bacteria, and the number of animals involved in the study. After that, 98 papers were included in the database. Figure 1 shows the PRISMA Flow Diagram illustrating the literature search, identification, screening, and assessment for eligibility leading to the final article selection.

## Non-*aureus* staphylococci and mammaliicocci

The *Staphylococcus* genus is a group of Gram-positive bacteria that comprises around 85 species and 30 subspecies (Phe 2020). *Staphylococcus* can be divided into two large groups according to the ability to coagulate blood plasma, and this genus can be divided into coagulase-positive *Staphylococcus* (SCP), where *S. aureus* is the most pathogenic species, causing infections in humans and animals and the group of non-*aureus* staphylococci and mamaliicocci (NASM) which lacks the coagulase gene (Michels et al. 2021). The NASM have traditionally been considered normal skin microbiota and are opportunistic bacteria that cause intramammary infections (De Visscher et al. 2017).

In many countries, NASM have become emerging pathogens and the main cause of mastitis (mainly subclinical), in cows, goats, and sheep (Persson et al. 2011). Some studies suggest that NASM infections may cause more severe damage to the mammary gland than previously thought. To improve our understanding of the effect of NASM on the mammary gland, so far, more than 50 species have been characterized as causing mastitis in dairy ruminants, and this has led to identifying species-specific virulence and pathogenesis factors related to NASM, as each species differs in its effects on milk production and somatic cell count (SCC), mode of transmission, potential reservoir, and susceptibility to antimicrobials, indicating that some species may be more pathogenic than others (Adkins et al. 2018a; Jenkins et al. 2019). Since the pathogenicity of these bacteria is not entirely clear, animal models represent an alternative for the study of these bacteria, but this is expensive, and an alternative to studying experimental mammary infections is the use of small ruminants, this knowledge will provide important insights that will improve preventive and therapeutic strategies (Lasagno et al. 2018).

**Fig. 2:**
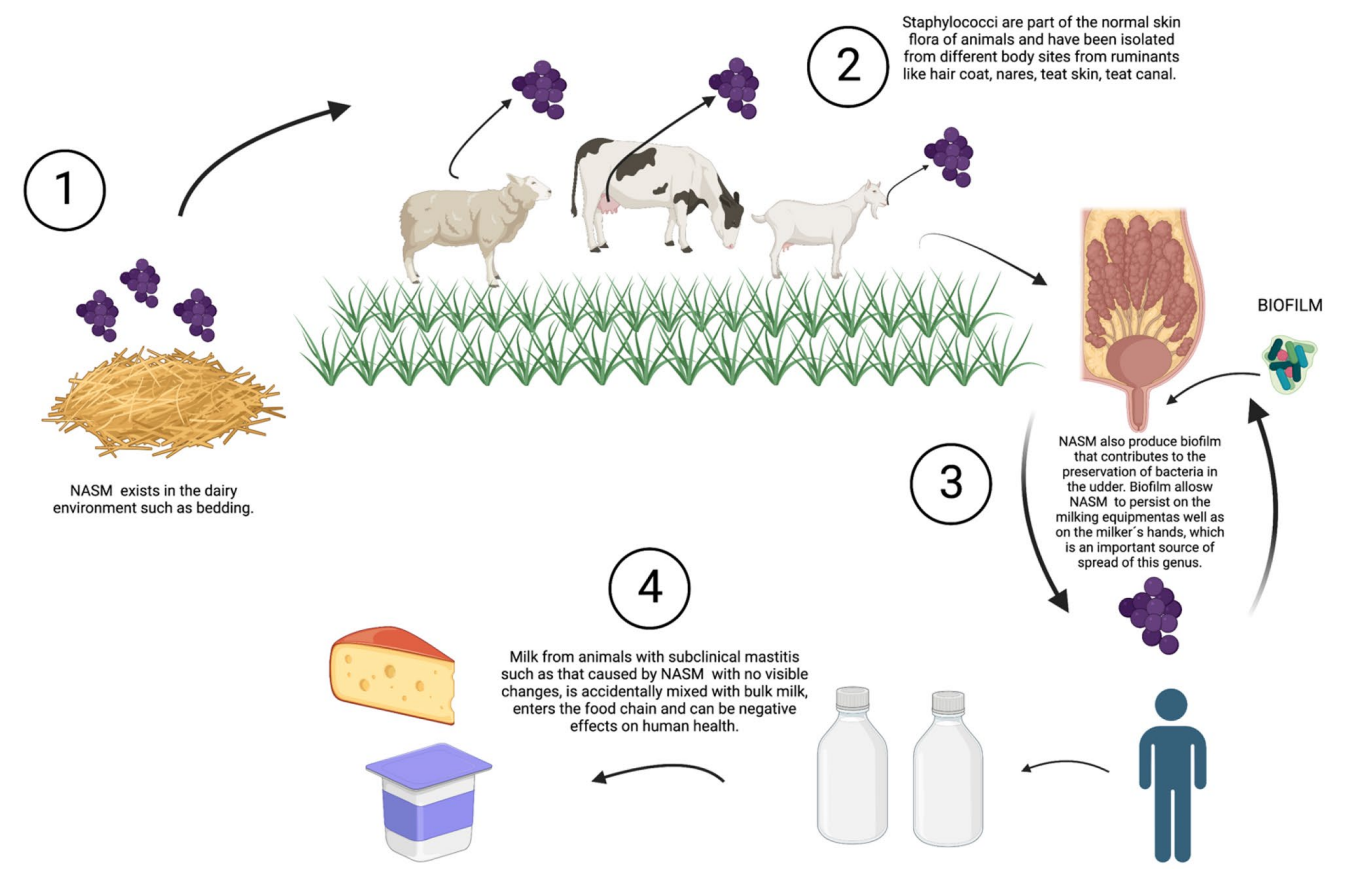
NASM environment, transmission, biofilm formation, and the effects on human health 1. NASM have been isolated from the dairy environment such as bedding as well as on the milker’s hands. 2. Staphylococci are part of the normal skin flora of animals and have been isolated from different body sites from ruminants like hair coats, nares, teat skin, and teat canals. 3. NASM produce biofilms that allow NASM to persist on the milking equipment as well as on the milker’s hands. 4. NASM have become emerging pathogens and the main cause of mastitis, mainly of the subclinical type when milk from animals with subclinical mastitis such as that caused by NASM with no visible changes, is accidentally mixed with bulk milk, enters the food chain and can be negative effects on human health

Non-*aureus* staphylococci (NAS) are abundant in dairy cows’ teat apices that are recovered from bovine fecal samples, and their differences in ecology, epidemiology, effect on udder health, and virulence or protective traits have been reported among other species within this group (De Visscher et al. 2016; Vanderhaeghen et al. 2015; Wuytack et al. 2020a, b). Several studies have reported that *S. chromogenes, S. epidermidis, S. xylosus*, *S. vitulinus*, *S. simulans*, and *M. sciuri* are the main NASM species isolated from the tips and skin of the animal’s teats increasing the probability to develop an intramammary infection during lactation, whereas in milk samples, *S. chromogenes*, *S. xylosus*, *S. haemolyticus* are most prevalent (Koop et al. 2012; Rosa et al. 2022; Ruiz-Romero et al. 2020; Traversari et al. 2019). Overall, more NASM isolates are identified in used bedding than in unused bedding (Adkins et al. 2022). Some species also produce a biofilm that allows NASM to persist on the milking equipment as well as on the milker’s hands, which is important for the spread of this genus (Pedersen et al. 2021; Silva et al. 2022) (Fig. 2). The prevalence and distribution of NASM are affected by environmental factors, geographic region, climate, water sources, access to pasture, type of production, and host factors such as the calving number and antimicrobials use (Taponen et al. 2009). In this context, it is important to determine the natural habitat of different NASM species, as this will define whether they should be considered environmental or host-adapted pathogens. This is also related to their commensal nature and their level of adaptation to the skin, the teat canal, and/or host udder (El-Jakee et al. 2013; Pyörälä et al. 2009).

Non-*aureus* staphylococci and mammaliicocci, potentially protect the udder against infection by major mastitis pathogens due to bacteriocin production, species such as *S. capitis, S. chromogenes, S. epidermidis, S. pasteuri, S. saprophyticus, S. sciuri, S. simulans, S. warneri*, and *S. xylosus* from bovine mammary glands are a source of potential bacteriocins, representing potential for future characterization and prospective clinical applications (Carson et al. 2017; Nascimento et al. 2005).

### Mastitis: economic impact

The incidence of clinical and subclinical mastitis during lactation varies greatly between herds, although subclinical mastitis is more prevalent than clinical mastitis. Due to variability in the intensity of detection at the herd level, the economic impact of subclinical infections is more difficult to quantify and predict between herds (Heikkilä et al. 2018; Hussein et al. 2022; Piepers et al. 2013). There is significantly more scientific evidence on the impact of clinical mastitis on health and productivity compared to subclinical mastitis (Rollin et al. 2015). Economic losses from mastitis include direct costs due to diagnostic tests, veterinary service, medication, and labor, as well as indirect costs associated with future loss of milk production, premature culling, and replacement of cows with mastitis. At the farm level, dairy farmers typically underestimate the costs of mastitis. However, long-term milk production shortfalls attributable to mastitis contribute to a notable portion of the economic losses in dairy systems (Heikkilä et al. 2018; Rollin et al. 2015; Piepers et al. 2010).

The costs of preventive measures must also be considered in the total costs of mastitis. The extent of the economic losses varies significantly between countries, depending on factors such as the milk price, treatment costs, and replacement of animals (Rollin et al. 2015).

Infections caused by NASM can cause mild inflammation in the mammary gland that results in a 3- to 4-fold increase in SCC, reducing both the quality and price of milk, however, SCC low in bulk milk, translates into an economic incentive for the producer to maintain and/or improve milk quality (Valckenier et al. 2020, 2021; Heikkilä et al. 2018; Tomazi et al. 2015). In a study where milk losses in dairy cows caused by subclinical mastitis caused by NASM at peak lactation were evaluated, a reduction of up to 1.8 kg/d of milk was detected, so NASM should not be underestimated as a cause of reduction of milk production (Heikkilä et al. 2018).

### Mastitis and Public health

Due to improved sanitation of milk production practices and milk heat treatments, the threat of various diseases and the incidence of outbreaks related to milk and dairy products have been greatly reduced in developed countries. However, a variety of microorganisms still contribute to disease outbreaks (Ibrahim et al. 2022; Hussein et al. 2022). In cases of severe clinical mastitis, milk abnormalities are easily observed, and the milk must be discarded so that milk would not normally enter the food chain. But when milk from animals with subclinical mastitis such as that caused by NASM with no visible changes, is accidentally mixed with bulk milk, it enters the food chain with potentially negative effects on human health (El-Jakee et al. 2013; Hussein et al. 2022). NASM can produce toxins and for this reason, the consumption of raw milk is not recommended, due to high contamination from animals, pastures, milking machines, and containers. To prevent human health problems, heat treatment is mandatory to ensure its safety and prolong its shelf life (Heikkilä et al. 2018). Although pasteurization is likely to destroy most pathogens, there is a concern when raw milk is consumed or when pasteurization is incomplete or faulty (Heikkilä et al. 2018). Despite the considerable advances that have been made to improve dairy food safety, there is rising concern that pasteurization is not sufficient for the destruction of plasmid-mediated antimicrobial resistance (AMR) genes of resistant bacteria and could stimulate bacteria to enter a viable but nonculturable (VBNC) state (Taher et al. 2020a, b). Toxins secreted by NASM are produced due to improper cooling of milk, during the manufacture of dairy products, and due to post-processing contamination, these toxins are not inactivated by extreme heat or cold and can cause food poisoning (Heikkilä et al. 2018; Hussein et al. 2022).

### Antimicrobial resistance in NASM

Antimicrobials are an important tool in mastitis control programs; therefore, surveillance of antimicrobial susceptibility is important to ensure optimal results when using them and minimize the risk of resistance (Bowler et al. 2020; Kizerwetter-Świda et al. 2018). Staphylococci can express resistance to a range of antimicrobials, of which methicillin resistance is of public health concern (Crespi et al. 2022; Fergestad et al. 2021). Infections caused by methicillin-resistant *Staphylococcus* (MRS) are more damaging due to the long-term treatments and limited drug options (Adkins et al. 2018b; Crespi et al. 2022; Mahato et al. 2017; Virdis et al. 2010). Recent reports from different parts of the world revealed that MRS are an emerging cause of infection and a potent threat to the dairy industry and public health due to their zoonotic potential (Kizerwetter-Świda et al. 2018; Windria et al. 2016). The increased exposure to drugs and the use of antimicrobials in animal diseases represents a danger to human health whose impact and effect are not yet well characterized but may lead to the emergence of antimicrobial-resistant strains, which makes antimicrobial susceptibility surveillance very crucial (Persson et al. 2011).

Another concern associated with NASM is that they may also act as reservoirs for antimicrobial resistance genes and may transfer resistance genes into the *S. aureus* genome, leading to the development of new multidrug-resistant strains (Persson et al. 2011), and the main reason for the increase in the rate of NASM infections is the spread of resistance to antimicrobials in this bacterial group (Persson et al. 2011). The production of beta-lactamases is the most common resistance mechanism in staphylococci, and its production is more common among subclinical NASM isolates than clinical isolates (De los Santos et al. 2022). NASM species may exhibit resistance to the following antimicrobials: penicillin, cefoxitin, erythromycin, clindamycin, gentamicin, streptomycin, tetracycline, ciprofloxacin, chloramphenicol, fusidic acid, oxacillin, vancomycin, and trimethoprim-sulfamethoxazole (Gurler et al. 2022; Ibrahim et al. 2022; Raspanti et al. 2016; Sawant et al. 2009).

### Non aureus staphylococci and mammaliicoccii as a cause of mastitis

Veterinary medicine interest in NASM has developed during the last 25 years, mainly due to the sharp increase in the rate of opportunistic infections, NASM generally have a beneficial relationship with their host and develop from commensals to pathogens only after damage to a natural barrier such as the skin, which can occur from a trauma or tissue injury (De Buck et al. 2021; Jenkins et al. 2019).

Pathogen colonization depends on adhesion factors, evasion of the host’s immune system, and the production of factors damaging to the host’s tissue, such as toxins and degradative exoenzymes. However, some virulence factors are found less frequently in NASM compared to *S. aureus*, making NASM infections more silent than *S. aureus.* (Adkins et al. 2018b; de Buck et al. 2021). Some NASM strains showed multiple virulence factors that are more likely to hydrolyze DNA, hemolysis, produce gelatinase and biofilm, and have multi-drug resistance as compared to other less virulent staphylococci (Zigo et al. 2022).

Bacteria adherence seems to be an essential first stage for the internalization of bacteria into the cytoplasm of the host cell, which is considered an important virulence strategy enabling bacteria to occupy a microenvironment separated from host defence mechanisms (Souza et al. 2016). The NASM species and strains adhered and internalized into MEC slower than did *S. aureus* (Souza et al. 2016). NASM can induce a mild local host response and animals infected with NASM show mild to moderate clinical signs of mastitis, differences in staphylococci species in evading phagocytosis and triggering ROS production, may explain the ability of some staphylococci species (*S. aureus* and *S. chromogenes*) to cause persistent infection and induce inflammation (Piccart et al. 2016; Simojoki et al. 2011; Souza et al. 2022).

In general, three groups of virulence factors are involved in the pathogenesis of staphylococcal infections; secreted proteins (cytotoxins, superantigens, and tissue-degrading enzymes), cell surface-bound proteins (microbial surface components that recognize adhesive matrix molecules), and cell wall components (polysaccharide capsule and lipoteichoic acid) (Zigo et al. 2022). It is important to argue deeply whether some NASM species should be considered true (minor) pathogens or harmless commensals (Otto 2004; Ruiz-Ripa et al. 2020).

To carry out specific studies for each species of NASM and the damage they cause in the mammary gland, it is necessary to carry out a correct identification of the isolates obtained from milk samples of animals with subclinical mastitis (Windria et al. 2016).

### Identification methods of non aureus staphylococci and mammaliicocci

NASM infections in dairy herds are generally managed as a single group without regard to different species, and most subclinical NASM infections go untreated (Turchi et al. 2020; Windria et al. 2016; Zaatout et al. 2019). The correct identification of the NASM species causing mastitis and possible persistent infections is important in providing arguments for the need for identification at species not only genus level (Capurro et al. 2009).

The following subsections will discuss the advantages and disadvantages of the different phenotypic and genotypic tests that have been used in various studies for NASM identification (Table 1).

**Table 1:** Advantages and disadvantages of phenotypic and genotypic identification

TYPING METHOD	ADVANTAGES	DISADVANTAGES		
**PHENOTYPIC**				
Biochemical test	The first step to enhance knowledge about the role of NASM, low cost, and easy procedures	Misidentifies a significant number of isolates		
API Staph	Easy procedure	Primarily designed to analyse biochemical reactions of human strains and not veterinary pathogenic strains		
**GENOTYPIC**				
Polymerase Chain Reaction (PCR)	Discriminatory power	Optimization of reaction conditions can be tedious and time-consuming for some PCR methods		
Pulse-field gel electrophoresis (PFGE)	Discriminatory power	Cost and reproducibility of results across different laboratories		
16 and 23 S rDNA sequencing	Provide a cost-effective technique to identify strains that may not be found using traditional methods	Absence of a database specific for the 16–23 S rRNA encoding region.		
Random amplified polymorphic DNA assay (RAPD)	Quick and easy to assay, low quantities of template DNA are required, RAPDs have a very high genomic abundance and are randomly distributed throughout the genome	Labor-intensive and time-consuming, low reproducibility, highly standardized experimental procedures are needed		
Restriction Fragment Length Polymorphism (RFLP)	High reliability, reproducible results, discriminatory results	Labor-intensive and time-consuming		
Amplified fragment length polymorphism (AFLP)	Discriminatory power, reproducibility, and type ability	Complex analysis and pure culture are required to prevent misinterpretation of results due to foreign DNA		
Ribotyping	Typeability	Discriminatory power, cost, and time		
DNA Hybridization	It can be applied to frozen tissues to enable maximum use of tissues that are difficult to obtain	For samples that have low DNA and RNA copies, it may be difficult to identify targets		
DNA sequencing	Characterise non-cultivable bacteria, profile hundreds of microorganisms from a single analysis, and provide faster and more accurate classification than traditional identification methods like cloning and culturing Identify low-abundance bacteria	High cost		
DNA Microarray analysis	Rapid and specific	Low signal intensity due to improper content of targeted DNA and probe can lead to inaccurate analysis		
MALDI-TOF MS	Fast, accurate, trained laboratory personnel are not required	The high initial cost of the MALDI-TOF equipment		

#### Phenotypic methods

Phenotypic characterization is based on data provided by all typing methods that do not analyze nucleic acids (Donelli et al. 2013). These tests are an important source of data for a preliminary description of taxa, from species to genera and families, in fact, in many cases, the set of all the morphological, physiological, and biochemical characteristics of a strain allows recognition of taxa (Donelli et al. 2013). The phenotypic characterization of the NASM was for many years, the only method of identification, for which many of the isolates obtained from the milk of ruminants with mastitis have been identified through phenotypic methods to characterize different species (Park et al. 2011; Vanderhaeghen et al. 2015).

Regarding laboratory diagnosis, microscopy is used by the Gram method to verify the bacterium’s morphology (Gram-positive cocci, arranged in grape-like clusters); novobiocin test to determine the susceptibility pattern of bacterium to antibiotic *novobiocin*; and the use of biochemical tests, such as coagulase and catalase tests to trace coagulase-negative strains. While identification is done with the use of selective and nonspecific culture media, such as blood agar (in which the hemolytic patterns of pathogens are observed), mannitol salt agar, and DNAse agar (selective to *S. aureus* strains, *S. intermedius*, and *S. hyicus*) (Moraes et al. 2021).

The use of phenotypic methods to differentiate NASM species can be seen as a first step to improving knowledge about the role of NASM, different methods have been used to phenotypically identify NASM, including a conventional identification scheme and commercial biochemical kits (Park et al. 2011).

A commercially available identification system is the API STAPH ID 20, which is recommended for the identification of NASM species isolated from intramammary infections by the National Mastitis Council (NMC) (Park et al.,2011). This method allows the study of carbohydrate metabolism and is capable to identify NASM species in 24 h (Park et al. 2011). The disadvantage of this identification system is that it is not designed to identify NASM from animal samples, so the database of phenotypic characteristics from animal NASM isolates is limited (Koop et al. 2012; Park et al. 2011; Vanderhaeghen et al. 2015).

Automated systems, such as Vitek® legacy (bioMérieux) and MicroScan® (Dade Behring), are also frequently used for rapid identification and antimicrobial susceptibility testing of Gram-positive cocci in clinical laboratories around the world, however, the automated system Vitek2 needs further improvement to provide reliable results for the characterization of the other NASM such as *S. epidermidis, S. cohnii, S warneri*, and *S. capitis* (Alves et al. 2009).

Some epidemiological studies in NASM have described inaccuracies in this type of identification due to the possibility of identifying NASM belonging to different species as the same microorganism, so estimates on the sensitivity, specificity, and true positive fraction of phenotypic tests are needed concerning genotypic tests (Adkins et al. 2018b). Phenotypic and genotypic data can contribute significantly to characterizing any NASM isolated at the species and subspecies levels (Jenkins et al. 2019; Onni et al. 2012). Diagnostic laboratories identify strains isolated by classical methods such as Gram stain and biochemical tests (i.e., catalase and coagulase detection) which represent a low cost, availability, and are easy to perform, however, there is a growing tendency for identification procedures to become polyphasic, that is, the “Polyphasic taxonomy” which is based on the integration of morphological, physiological, biochemical, and molecular characteristics without neglecting the need to develop rapid and low-cost diagnostic tests to identify the different NASM species (Donelli et al. 2013). Currently available molecular techniques provide an important contribution to identifying and classifying microorganisms based on their genotypic characteristic (Donelli et al. 2013). In the next section, there is a resume of the most common genotypic techniques to identify NASM.

#### Genotypic methods

The application of molecular techniques has greatly improved the ability to correctly identify bacterial isolates and their classification, particularly genotypic methods directed toward DNA or RNA molecules. Since phenotypic methods to identify NASM from the milk of ruminants often yield unreliable results, methods for molecular identification based on gene sequencing or fingerprinting techniques have been developed (Vanderhaeghen et al. 2015). The use of molecular identification methods has shown diversity among the NASM species that can be found in herds, as well as differences between species regarding intramammary infections, persistent infections in the mammary gland, and ecological niches inside and outside the animal’s body (Vanderhaeghen et al. 2015). Molecular characterization of antimicrobial resistance genes on farms and in commercial milk with emphasis on the effect of currently practiced heat treatments on the viable but nonculturable formation (Braem et al. 2011).

Molecular tests specifically identify isolates at the species level and help increase our understanding of important NASM species isolated from clinical samples. Therefore, the results of such tests can help to make decisions on the use of specific treatments to eliminate persistent infections, implement control measures, and/or improve milking practices (Donelli et al. 2013).

Table 2 shows the NASM species recovered from cows, goats, and sheep milk with mastitis that were identified with different phenotypic and genotypic techniques globally since the beginning of the 2000s. It highlights the fact that the reports of isolated species of NASM in sheep are much lower worldwide than that reported in cows. This may be because the cow’s dairy industry is the most important worldwide and most studies to reduce cases of mastitis, are directed towards this species to reduce cases of mastitis, leaving goats and sheep in second and third place respectively, even though the dairy products of these two species are increasing as reported in recent years. However, the NASM species reported in these three species are similar, reporting *S. chromogenes* as the most common NASM recovered from the milk of animals with mastitis (FAO 2020; Pyörälä et al. 2009).

**Table 2:** Identification techniques used for NASM around the world

YEAR	COUNTRY	ANIMAL	SPECIES-NO. OF CASES	IDENTIFICATION METHOD	REFERENCE	
**2005**	Turkey	cows	*S. hyicus 20, S. chromogenes 16, S. epidermidis 9, S. haemolyticus 5, S. sciuri 4, S. lentus 3*	Biochemical test/phenotypic identification	Kirkan S et al. 2005	
**2013**	Mexico	goats	*S. chromogenes 30, S. xylosus 14, S. sciuri 2, S. haemolyticus 1. S. caprae 1, S. epidermidis 1, S. cohnii 3*	API Staph/phenotypic identification	Ruiz-Romero 2018	
**2014**	Uganda	cows	*S. epidermidis 45, S. haemolyticus 8*	Biochemical test/phenotypic identification	Björk et al. 2014	
**2016**	Indonesia	goats	*S. pasteuri 3, S. xylosus 5, S. haemolyticus 5*	Polymerase Chain Reaction (PCR)/genotypic identification	Windria et al. 2016	
**2019**	United States of America	cows	*S. chromogenes 290, S. haemolyticus 108, S. simulans 39, S. S. epidermidis 39, S. hominis 28, S. auricularis 25, S. sciuri, 13, S. capitis 11, S. cohnii 9, S. warnerii 6, S. pasteuri 5, S. xylosus 4, S. hyicus 3, S. equorum 2, S. microti 2, S. rostri 2, S. gallinarum 1, S. saprophyticus 1, S. succinus 1.*	DNA sequencing/genotypic identification	Jenkins et al. 2019	
**2019**	Algeria	cows	*S. sciuri 38. S. xylosus 20, S. succinus 6, S. lentus 6, S. hominis 5, S. warneri 5, S. capitis 4, S. saprophyticus 4, S. caprae 2, S. lugdunensis 2, S. simulans 2, S. chromogenes 1, S. equorum 1, S. haemolyticus 1.*	Microarray analisis/genotypic identification	Zaatout et al. 2019	
**2020**	Italya	ewes	*S. epidermidis 11, S. simulans 3, S. chromogenes 1, S. caprae 1, S. arlettae 1, S. jettensis 1. S. haemolyticus 1, S. xylosus 1.*	PCR-RFLP/genotypic identification	Turchi et al. 2020	
**2021**	Ethiopia	cows	*S. sciuri 8, S. lentus 2*	Biochemical test/phenotypic identification	Dabele et al. 2021	
**2021**	Colombia	cows	*S. epidermidis 87, S. chromogenes 115, S. sciuri 65, S. simulans 76, S. haemolyticus 58, S. capitis 70, S. hominis 34, S. xylosus 23, S. equorum 28, S. auricularis 32, S. hyicus 36*	Pulse-Field Gel Electrophoresis/genotypic identification	Andrade-Becerra et al. 2021	

## Biofilm formation

Antimicrobial tolerance has been defined as the ability of bacteria to survive exposure to antimicrobials without developing resistance. It has also been reported that tolerance invariably precedes antimicrobial resistance, indicating that preventing tolerance may offer a new perspective for controlling antimicrobial resistance (Kranjec et al. 2021). Whereas antimicrobial resistance is genetically induced through mutations or horizontal gene transfer, antimicrobial tolerance involves bacterial survival through phenotypic states of biofilms and persistent dormant cells (Bowler et al. 2020).

Biofilms are communities of microorganisms, which are bacteria that live naturally and preferentially as communities adhered to an inert surface or in living tissues. Once adhered, the bacterial cells establish and organize themselves within a self-produced extracellular polymeric substance to form a matrix that protects against environmental threats, thus providing an extremely effective survival strategy (Bowler et al. 2020; Kranjec et al. 2021; Silva et al. 2022).

The formation of biofilms in animals with mastitis is considered a selective advantage for pathogenic microorganisms, which contributes to the preservation of bacteria in the udder (Kranjec et al. 2021). Biofilm can be detected using Congo Red Agar, congo red binds to exopolysaccharides present in the biofilm, however, compared to other methods, it is the least recommended due to high false negatives, while a positive test is evidenced if crystalline colonies of black color and dry appearance are observed (Darwish et al. 2013). Biofilm formation can also be detected by targeting some biofilm-associated genes such as *icaA, bap*, *aap*, *embP*, *fbe, atlE*, and *eno* that are present in some NASM isolates such as *S. chromogenes, S. epidermidis, S. devriesei, S. xylosus and S. haemolyticus* in animals with clinical or subclinical mastitis (Srednik et al. 2017). Biofilm formation and the presence of various staphylococcal virulence factors do not seem to directly influence the effect of NASM on IMI, but the available information is indirect or insufficient to draw consistent conclusions (Vanderhaeghen et al. 2014b). Biofilm is an important virulence factor in mastitis and as a result, infections become more difficult to treat and eradicate. In fact, in dairy animals with mastitis, biofilms within the udders reduce the effect of antimicrobials and allow microorganisms to evade the innate immune system (Lee et al. 2022; Pedersen et al. 2021). Some *S. chromogenes* strains have been confirmed to display a non-biocidal inhibition of pathogenic biofilms and were able to significantly inhibit *S. aureus* and NASM biofilm formation in a dose-independent manner and without affecting the viability of bovine cells, these findings reveal a new activity of the udder microflora of healthy animals (Beuckelaere et al. 2021; de Vliegher et al. 2004; Isaac et al. 2017; Toledo-Silva et al. 2022).

Biofilm is one of the main causes of developing chronic infections, biofilm development is a complex process that involves many staphylococcal proteins that can be divided into three general stages: attachment, multiplication/maturation, and shedding/dispersion (Bowler et al. 2020).

Biofilm-associated infections are often treated with conventional antimicrobials. However, after diagnosis of staphylococcal infections, therapeutic options are often limited due to the widespread resistance mechanisms developed by these bacteria. The high propensity of staphylococci to form biofilms confers additional competitive advantages, including a 10- to 1,000-fold increase in resistance/tolerance to antimicrobials, increasing their resilience to treatment (Kranjec et al. 2021; Pascu et al. 2022).

The extracellular matrix of biofilms can act as a shield, effectively hindering the penetration of antimicrobials into biofilms. This makes it difficult for many antimicrobials to reach the cells present in the deeper layers, leading to heterogeneous exposure to antimicrobials in biofilms. Cells in the deeper layers of biofilms can be exposed to subinhibitory concentrations of antimicrobials until they potentially encounter a lethal dose (Wei et al. 2022). This gradual exposure activates the transcriptional response at low doses, potentially promoting antimicrobial resistance mechanisms (Pascu et al. 2022; Wei et al. 2022). Therefore, care should be taken when treating biofilms with antimicrobials with high resistance potential. This can be achieved by choosing agents characterized by modes of action that reduce the likelihood of resistance development or by adopting combined therapies (i.e., multiple antimicrobial agents with different modes of action) to treat infections (Wei et al. 2022).

### Biotechnology to eliminate biofilms

The spread of antimicrobial-resistant pathogens that normally survive in biofilms such as NASM and the recent COVID-19 pandemic (although they are unrelated phenomena) have demonstrated the urgency and need to discover new methods to combat these growing threats (Abdullahi et al. 2016). The combination of antimicrobials with nanocarriers, ultrasound, electric current, phage therapy, and drug delivery system, has shown promising results (Abdullahi et al. 2016).

The new alternatives to prevent, treat and eradicate the biofilm and the bacteria found within it, have been used mainly in human medicine, while for veterinary medicine, the information on new biotechnologies to prevent the formation of biofilms in NASM is scarce. These alternatives can be adapted to dairy farms and monitor herds to be able to reduce the incidence of mastitis caused by NASM and avoid chronic infections due to the presence of biofilms (Table 3).

**Table 3:** Alternatives to control, treat and eliminate biofilms

GOAL	AVAILABLE OPTIONS	TARGET/MECHANISM OF ACTION	REFERENCES
**PREVENTION OF BIOFILMS FORMATION**	Silver and copper ions	Antibacterial properties.	Lison et al. 2022
	Surface modification	a. Titanium implant modification´s impact on improved biocompatibility and prevention of bacteria adhesion and biofilm formation.	
		b. Nanoparticles like zinc-oxide, silver, or polyethyleneimine are added to composite material to stop the increasing of bacteria by blocking the cell wall function, inhibiting active transport and metabolism of sugar, displacement of magnesium ions necessary for the enzyme bacterial biofilm activity, and preventing DNA replication.	
	Anti-quorum-sensing agents	a. The loss of AIP* and RNAIII production affects biofilm formation in *S. aureus*. As such, blocking signal production or degrading the signal might be promising strategies	Brackman et al. 2015
		b. Block AI-2 production.	
**DETECTION OF BIOFILMS**			
Biofilm sensors	Sensor-less Microfluidic systems	pH, flow rates, and temperature.	Subramanian 2020
	Optical systems	Oxygen. pH, ions, temperature, metabolites.	Funari et al. 2022
	Electrochemical systems	Oxygen, pH, ions, temperature, metabolites.	
	Mechanical systems	Interfacial material, parameters for adhesion properties.	
**TREATMENT OF BIOFILMS**			
Antimicrobial with devices and therapeutic options	Ultrasound	This device enhances the bactericidal action of the antimicrobial agent, through the passage of non-invasive acoustic energy waves through the skin to the site of biofilm.	Abdullahi et al. 2016
	Electric current	Synergetic use of low-level electric current with antimicrobials enhances the antimicrobial activity of antimicrobials which ordinarily are resisted by biofilm organisms.	
	Phage therapy	The mechanism through which phage achieves its antibiofilm action is by enzyme production which hydrolyses and degrades the extracellular matrix of biofilm, perhaps, the use of bacteriophage or combination with antimicrobial will be effective.	
	Drug delivery system	This system involves a combination of antimicrobial drugs with nano-carriers. Antimicrobials such as gentamycin, ampicillin, and ciprofloxacin among others are encapsulated in a drug delivery nano-carrier. Examples of commonly used nano-carriers include phosphotydyl-choline, polyethylene glycerol, polyamidoamine, and polyacrylate.	
	Bacteriocins	Disruption of the membrane integrity on target cells leads to leakage of the cell contents and membrane potential dissipation, and ultimately cell death.	Kranjec 2021
	Phage-Derived Antibiofilm Strategies	Kill their bacterial host at the end of the lytic cycle.	
	Antibodies	Disrupting staphylococcal biofilms by using antibodies targeting different staphylococcal antigens, such as surface proteins, cell-wall enzymes, PNAG, and toxins.	
	Photoinactivation	Photodynamic inactivation (PDI), also known as photodynamic therapy, is based on the use of visible light, a photosensitizer, and oxygen to produce a phototoxic reaction that kills bacteria.	
	Nanotechnology	The use of nanoparticles with antimicrobial activity and the development of drug delivery systems.	Pinto 2019
	*a) Lipid-based formulation*	Liposomes, quatsomes, solid lipid nanoparticles, micelles, nanodroplets	
	*b) Polymeric nanoparticles*	Polymeric NPs are composed of biodegradable polymers and are characterized by high structural integrity.	
	*c)Metallic nanoparticles*	Metallic NPs can be applied as drug delivery systems once they can protect the drugs until their target site and avoid immune system activation with low cytotoxicity.	
	*d) Magnetic nanoparticles*	Potential targeted nanosystems as they can be directed or guided by magnetic field gradient toward biological targets.	
	*e) Silica-bases nanosystems*	Mesoporous silica nanoparticles are promising drug delivery systems due to their biocompatibility and their stability that leads to a controlled drug release.	

*AIP autoinducing peptide, ^+^AI-2 autoinducer 2

The following section describes some of the latest technologies being used to combat biofilms.

#### Prevention of biofilm formation

The best universal way to prevent the development of bacterial biofilms has not been discovered. Yet, nowadays, there is a plethora of antibacterial coating technologies (Brackman et al. 2015; Lison et al. 2022). Coating technology can be classified into three groups. The first is passive surface finish modification (PMS), which focuses on preventing and reducing bacterial adhesion without releasing germicide to surrounding tissues (Lison et al. 2022). Active surface finishing/modification (ASM) is the next way to create ideal coatings. This type of layer includes pharmacologically active agents, germicides, metal ions, and different organic or inorganic ingredients (Brackman et al. 2015). Finally, there are local perioperative antibacterial carriers or coatings (LCC), that allow the use of biodegradable or non-biodegradable antibacterial coatings or carrier concentrations (Lison et al. 2022).

To prevent the development of biofilms and bacterial growth, silver and copper ions can be used, and surface modification using nanoparticles like zinc-oxide, silver, or polyethyleneimine added to composite compounds. These positively charged metal ions adhere to the negatively charged bacterial cell wall and cause cell lysis and death. There is an urgent need for long-term active coatings that work against multiple bacterial strains and drug-resistant strains (Brackman et al. 2015; Lison et al. 2022).

Another reported way to avoid the formation of biofilms is by using anti-quorum-sensing (QS) agents. QS is a process by which bacteria produce and detect signal molecules and thereby coordinate their behavior in a cell-density-dependent manner. In this way, they can be used as target molecule Autoinducer Peptide (AP) and Autoinducer 2 (AI-2) block this communication and prevent the formation of biofilms (Brackman et al. 2015).

Before they can be used in practice, more research is needed to investigate the involvement of quorum-sensing inhibitors (QSI) in biofilm formation, maintenance, and dispersal, and to develop several more active non-toxic QSI. Despite this, QSI are promising antibiofilm agents and may be of great value in the future treatment of bacterial infections (Brackman et al., 2015).

The isolated microorganisms from clinical and subclinical mastitis exhibited different and highly heterogeneous sensitivity to the action of antimicrobial drugs. This causes difficulty in the effective control of mastitis in ruminants, which is frequently caused by pathogenic associations of microbial biofilms. Therefore, to combat this syndrome, it is important to explore these alternatives (Rudenko et al. 2021).

#### Biofilm sensors

Due to their strong adhesion on the surface, microbial biofilms can also be utilized positively as sensing elements in cell-based sensors. This characteristic provides an opportunity to take advantage of the unusual properties of biofilms to develop biofilm-based sensors where the biofilm itself serves as the sensing element responsible for detection (Funari et al. 2022). These types of cell-based biosensors are cheap and robust, require little maintenance, and can operate continuously for a long time, making them favorable sensing devices for remote areas and online monitoring. They have been used to measure indicators of water pollution and have been deployed as early warning devices to capture the release of toxic compounds into the aqueous environment, which can alert authorities to take immediate action (Subramanian et al. 2020). This technology can be adapted and applied in dairy farms since biosensors can be compatible with milk. Detection of the formation of biofilms in milk storage tanks or in equipment used for milking such as teat cups, will allow for early detection and prevent the development of serious intramammary infections (Subramanian et al. 2020).

To bring this technology to the field of veterinary medicine, greater synergy between microbiologists, biotechnologists, chemists, scientists, and engineers is required to develop new biofilm-related sensors for many cross-cutting applications (Funari et al. 2022; Subramanian et al. 2020).

#### Treatment of biofilms

Effective treatment of biofilm infection requires a dual approach through a combination of antibiofilm and antimicrobial agents (Pinto et al. 2019). The pathophysiology of biofilm infection is thought to be regulated by the quorum sensing mechanism, through a cascade of events in which a community of microorganisms, united as a single entity expresses gene virulence, and antimicrobial properties (Kranjec et al. 2021). The conventional antimicrobial approach has a restricted range of action against fast-growing pathogenic organisms with little or no effect on biofilm. However, more radical therapeutic approaches involve the combination of conventional antimicrobials with methodologies like ultrasound, electric current, phage therapy, and drug delivery system described (Table 2) (Abdullahi et al. 2016; Kranjec et al. 2021; Pinto et al. 2019). These technologies have been applied mainly to MRSA as it is one of the most important pathogens causing hospital infections. All these technologies must be used and applied in dairy farms, both in milking machines and storage tanks, as well as adaptations to the treatments that are used in animals to prevent the formation of biofilm leading to chronic mastitis, ultimately avoiding economic losses (Kranjec et al. 2021; Pinto et al. 2019).

Treatment of staphylococcal biofilm infections is very challenging. Biofilm-associated staphylococci have reduced susceptibility to antimicrobials because of a protective biofilm matrix and phenotypic heterogeneity in the biofilm population, including non-growing and slow-growing cells (Abdullahi et al. 2016; Pinto et al. 2019). Some combination therapies re-sensitize pathogens to certain antimicrobials to which they have become resistant when given as monotherapies (Pinto et al. 2019). The molecular mechanisms behind it are not yet well understood, but this may be an invaluable approach to repurposing of many antimicrobials that have been rendered useless due to antimicrobial resistance, commonly seen with these types of multi-resistant NASM strains causing mastitis (Pinto et al. 2019).

## Challenges and opportunities

This section will describe some challenges and opportunities that authors consider to be necessary to fight infections caused by NASM and prevent intramammary infections in dairy animals by impeding the formation of biofilms that cause chronic infections. For that, it is necessary to continue developing biotechnology that detects the initial formation of biofilms and treats them with alternative therapies to antimicrobials.

### Pathogenesis of NASM as a cause of mastitis

Although some species of NASM involved in intramammary infections have more virulence factors compared to other species, little is known about the pathogenesis of each of the species within the mammary gland. For economic reasons, most studies have been carried out using goats. It is easier to carry out experimental infections in small ruminants. In vitro infections have been carried out that provide information on the damage that NASM can cause in mammary glandular tissue. It is necessary to start investigating the specific pathogenesis of each species in the mammary gland and to evaluate the damage caused and the consequences that these damages imply in terms of economic losses and public health risks (Lasagno et al. 2018; Ruiz-Romero et al. 2020).

### Identification of NASM

NASM are no longer considered a homogeneous group of bacteria and it is known that some species are more common in cases of ruminant mastitis than others. Phenotypic identification remains the first step for identifying this bacterial genus, however, it is not designed to accurately identify species, and it is important to differentiate between NASM species that are true pathogens or harmless commensals of the udder. The use of molecular techniques can lead us to this information. Therefore, the identification must be carried out integrally, combining several techniques until a polyphasic identification is made that, in turn, is easily accessible and reproducible (Donelli et al. 2013; Piessens et al. 2012; Vanderhaeghen et al. 2015). These strains of staphylococci with zoonotic potential have a high capacity to form biofilm and can represent a threat to human health. Therefore, this underlines the need to implement new measures in the veterinary and public health sectors to prevent NASM transmission in the context of One-Health (Abdullahi et al. 2016; Jacques et al. 2022).

#### Alternative to antimicrobials to treat cases of mastitis

In practice, the treatment received by animals is indistinct from the isolated species, and the indiscriminate use of antimicrobials to treat animals with mastitis without adequately identifying the genus and bacterial species has led to the generation of NASM strains multi-resistant to antimicrobials (Kranjec et al. 2021).

The frequent failures in the treatment of human and animal infections with antimicrobials give little reason for optimism regarding the prospects for this type of therapy. In summary, clinical practice has shown that systemic antimicrobials are not capable of providing effective treatment for these infections (Sandholm et al., 1990). Therefore, the investigation of new classes of antimicrobials or alternatives to antimicrobials such as the use of biosurfactants, phytochemicals, antimicrobial peptides, and microbial enzymes is urgently needed.

### Biofilms and new alternatives to avoid them

An alternative to the conventional antimicrobial strategy is the inhibition of biofilm formation. Antibiofilm strategies generally do not inhibit bacterial growth and division, but instead target the molecules and pathways involved in the formation and maturation of biofilms such as quorum-sensing, consequently, the selection pressure for the development of bacteria will be reduced antimicrobial resistance (Funari et al. 2022). In addition, factors involved in staphylococcal biofilm formation are highly species-specific (compared to the targets of conventional antimicrobials), and antibiofilm strategies may allow the development of narrow-spectrum precision agents that will have little or no effect on another microbiota (Funari et al. 2022; Subramanian et al. 2020).

## Final remarks

**Fig. 3:**
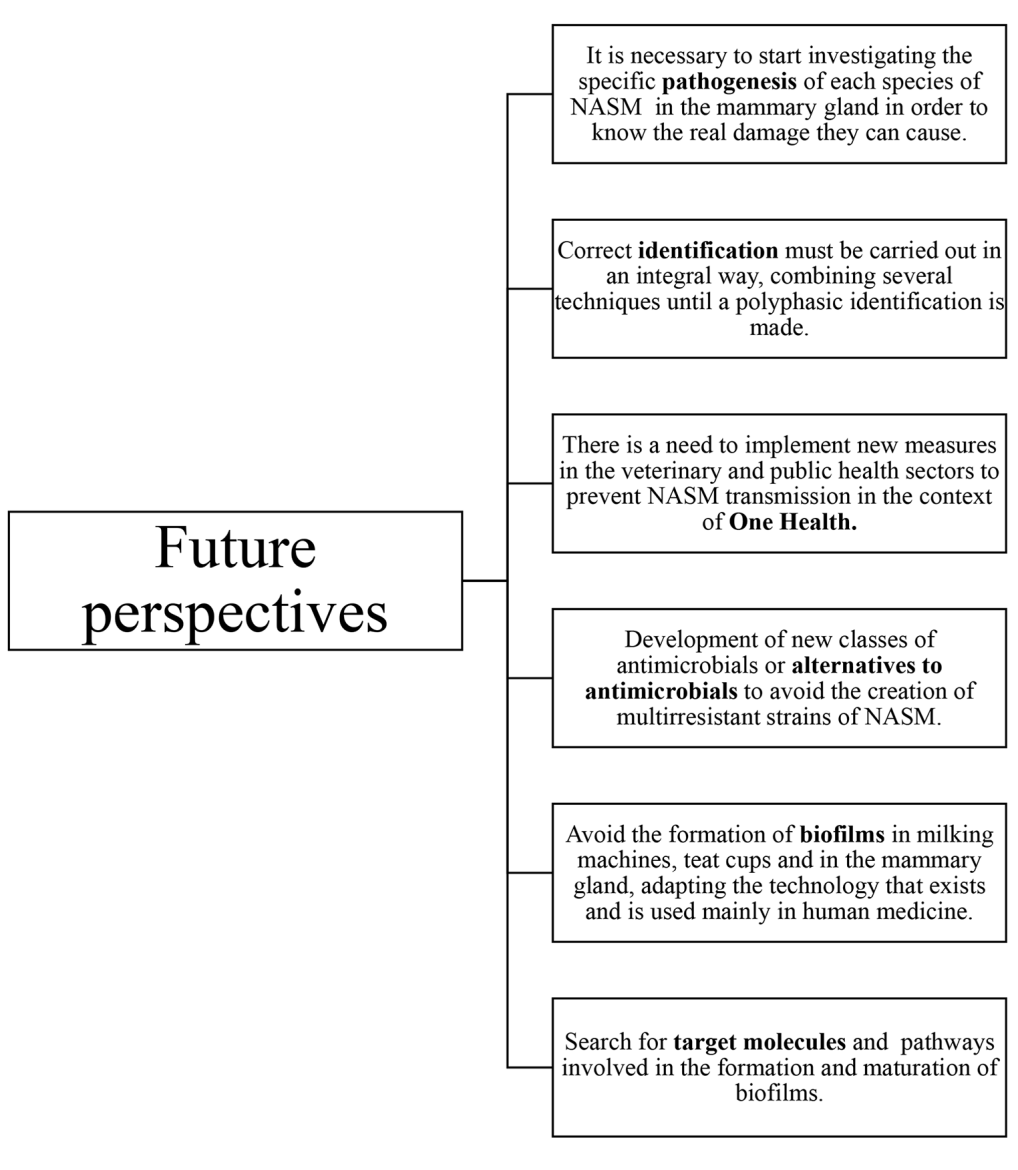
Future perspectives in the prevention of mastitis caused by NASM and treatment of biofilms

Overall, the following points should be considered for future research on Non-*aureus* staphylococci and mammaliicocci as a cause of mastitis in ruminants:

- Since the pathogenicity of NASM is not entirely clear, animal models represent an alternative for the study of these pathogenic bacteria. In terms of animal models, using cows is expensive, and the use of small ruminants could be an alternative for studying mammary infections.
- To prevent and control mastitis in ruminants, correct identification of the bacterial agent must be carried out, combining phenotypic and genotypic techniques whenever possible before using any type of antimicrobial agent to reduce the appearance of multiresistant bacterial strains, since there is a stage of tolerance to antimicrobials in which bacteria can develop biofilms before multiresistant bacteria appear.
- Regarding the control of the NASM, precise diagnostic techniques must be available to identify the bacteria at the species level. In developed countries, these techniques are easily accessible, but this is not the case in developing countries, since there are no diagnostic laboratories that have molecular identification techniques, therefore, the correct identification is still distant in these places.
- Formation of biofilms must be avoided by adapting technologies that are currently used in human medicine, that have the potential to be used in dairy farms, where the different technology can be adapted to avoid the formation of these bacterial communities, which would be the main thing instead of facing established biofilm communities that are more difficult to eliminate.
- Technologies that can be used to prevent the formation are the use of silver and copper ions to prevent bacteria from adhering and reproducing, as well as the use of sensors in milk tanks since they are effective and low cost. The use of these techniques will improve control of the disease, which translates into fewer sick animals and therefore less use of antimicrobials and a decrease in the appearance of multiresistant NASM.
- Anti-biofilm strategies do not inhibit bacterial growth and division, but instead target molecules and pathways involved in the formation and maturation of biofilms without necessarily killing biofilm-associated cells. Anti-biofilm strategies may allow for the development of narrow-spectrum precision agents, which will have low or no influence on other microorganisms. Therefore, the selection pressure for antimicrobial-resistance development will be lowered.
- The application of novel therapeutic approaches, such as phage therapy and the use of some mucolytic agents that are capable of inhibiting biofilm formation, is strongly recommended, and the dairy industry can reduce economic losses due to mastitis, if they can adapt this type of therapy.
- Even though research on the development of biofilms is mainly focused on human medicine, this technology must be developed at the same time in veterinary medicine, especially in the dairy industry where intramammary infections are extremely common causing economic losses and most importantly, these bacteria are capable of being transmitted to humans, so these investigations must be developed under the concept of one-health (Fig. 3).

So far, NASM are not given the importance they deserve, although it can cause subclinical mastitis in domestic ruminants. They are capable of persisting in the mammary gland, developing multi-resistance to antimicrobials, and are biofilm producers, which gives them the advantage of establishing themselves in the mammary gland and milking equipment. If there is already technology to eliminate, prevent and detect them in human medicine, why has not this technology been applied in veterinary medicine? Answering this would help to adapt technology that would reduce costs and incidence of mastitis and therefore prevent zoonotic infections and foodborne problems.

## Data Availability

Not applicable.

## References

[CR1] Abdullahi UF, Igwenagu E, Mu´azu A, Aliyu S, Umar MI (2016). Intrigues of biofilm: a perspective in veterinary medicine. Vet World.

[CR2] Adkins PRF, Dufour S, Spain JN, Calcutt MJ, Reilly TJ, Stewart GC, Middleton JR (2018). Cross-sectional study to identify staphylococcal species isolated from teat and inguinal skin of different-aged dairy heifers. J Dairy Sci.

[CR3] Adkins PRF, DufourS, Spain JN, Calcutt MJ, Reilly TJ, Stewart GC, Middleton JR (2018). Molecular characterization of non-*aureus Staphylococcus* spp. from heifer intramammary infections and body sites. J Dairy Sci.

[CR4] Adkins PRF, Placheta LM, Borchers MR, Bewley JM, Middleton JR (2022). Distribution of staphylococcal and mammaliicoccal species from compost-bedded pack or sand-bedded freestall dairy farms. J Dairy Sci.

[CR5] Alves D, Siquiera P, Gugel I, Lúcia J, Antunes A, Secchi S, Pasternak C, Dalla J, Martino M (2009). Evaluation of the Automated System Vitek2 for identification and Antimicrobial susceptibility testing of brazilian gram-positive Cocci strains. Braz J Infect Dis.

[CR6] Andrade-Becerra RJ, Tarazona-Manrique LE, Vargas-Abella JC (2021) Prevalencia y efecto de la infección intramamaria debida a especies de estafilococos coagulasa negativo (ECN) en el conteo de células somáticas en leche de vacas holstein en Boyacá, Colombia. Rev Med Vet Zoot 68(2). 10.15446/rfmvz.v68n2.98024

[CR7] Beuckelaere L, De Visscher A, Souza FN, Meyer E, Haesebrouck F, Piepers S, De Vliegher S (2021). Colonization and local host response following intramammary *Staphylococcus chromogenes* challenge in dry cows. Vet Res.

[CR8] Björk S, Båge R, Kanyima BM, André S, Nassuna-Musoke MG, Owiny DO, Persson Y (2014) Characterization of coagulase negative staphylococci from cases of subclinical mastitis in dairy cattle in Kampala, Uganda. Ir Vet J 67(1). 10.1186/2046-0481-67-12 10.1186/2046-0481-67-12 PMC4051667 24917926

[CR9] Bowler P, Murphy C, Wolcott R (2020). Biofilm exacerbates antibiotic resistance: is this a current oversight in antimicrobial stewardship?. Antimicrob Resist Infect Control BMC.

[CR10] Brackman G, Coenye T (2015). Quorum sensing inhibitors as Anti-Biofilm Agents. Curr Pharm Des Curr Pharm Des.

[CR11] Braem G, de Vliegher S, Supré K, Haesebrouck F, Leroy F, de Vuyst L (2011). (GTG)5-PCR fingerprinting for the classification and identification of coagulase-negative *Staphylococcus* species from bovine milk and teat apices: a comparison of type strains and field isolates. Vet Microbiol.

[CR12] Capurro A, Artursson K, Waller KP, Bengtsson B, Ericsson-Unnerstad H, Aspán A (2009). Comparison of a commercialized phenotyping system, antimicrobial susceptibility testing, and *tuf* gene sequence-based genotyping for species-level identification of coagulase-negative staphylococci isolated from cases of bovine mastitis. Vet Microbiol.

[CR13] Carson DA, Barkema HW, Naushad S, De Buck J (2017). Bacteriocins of non-*aureus* staphylococci isolated from bovine milk. AEM.

[CR14] Cervinkova D, Vlkova H, Borodacova I, Makovcova J, BabakV, Lorencova A, Vrtkova I, Marosevic D, Jaglik Z (2013). Prevalence of mastitis pathogens in milk from clinical healthy cows. Vet Med.

[CR15] Condas LAS, De Buck J, Nobrega DB, Carson DA, Roy JP, Keefe GP, DeVries TJ, Middleton JR, Dufour S, Barkema HW (2017). Distribution of non-aureus staphylococci species in udder quarters with low and high somatic cell count, and clinical mastitis. J Dairy Sci.

[CR16] Contreras A, Sierra D, Sánchez A, Corrales JC, Marco JC, Paape MJ, Gonzalo C (2007). Mastitis in small rumiants. Small Rumin Res.

[CR17] Crespi E, Pereyra AM, Puigdevall T, Rumi MV, Testorelli MF, Caggiano N, Gulone L, Mollerach M, Gentilini ER, Srednik ME (2022). Antimicrobial resistance studies in staphylococci and streptococci isolated from cows with mastitis in Argentina. J Vet Sci.

[CR18] Dabele DT, Borena BM, Admasu P, Gebremedhin EZ, Marami LM (2021). Prevalence and risk factors of mastitis and isolation, identification and antibiogram of *Staphylococcus* species from mastitis positive zebu cows in Toke Kutaye, Cheliya, and Dendi Districts, West Shewa Zone, Oromia, Ethiopia. Infect Drug Resist.

[CR19] Dalanezi F, Joaquim S, Guimaraes AF, Guerra S, Lopes B, Schmidt E, Cerri R, Langoni H (2020). Influence of pathogens causing clinical mastitis on reproductive variables of dairy cows. J Dairy Sci.

[CR20] Darwish SF, Asfour HAE (2013). Investigation of biofilm forming ability in staphylococci causing bovine mastitis using phenotypic and genotypic assays. Sci World J.

[CR21] De Buck J, Ha V, Naushad S, Nobrega DB, Luby C, Middleton JR, de Vliegher S, Barkema HW (2021). Non-*aureus* staphylococci and bovine Udder Health: current understanding and knowledge gaps. Front Vet Sci.

[CR22] De los Santos R, González-Revello A, Majul L, Umpiérrez A, Aldrovandi A, Gil A, Hirigoyen D, Zunino P (2022) Subclinical bovine mastitis associated with *Staphylococcus s*pp. in eleven Uruguayan dairy farms. *J* Infect Dev Ctries 6: 630–637. 10.3855/jidc.12960 10.3855/jidc.12960 35544624

[CR23] De Visscher A, Piepers S, Haesebrouck F, de Vliegher S (2016). Teat apex colonization with coagulase-negative *Staphylococcus* species before parturition: distribution and species-specific risk factors. J Dairy Sci.

[CR24] De Visscher A, Piepers S, Haesebrouck F, Supré K, de Vliegher S (2017). Coagulase-negative *Staphylococcus* species in bulk milk: prevalence, distribution, and associated subgroup- and species-specific risk factors. J Dairy Sci.

[CR25] De Vliegher S, Opsomer G, Vanrolleghem A, Devriese LA, Sampimon OC, Sol J, Barkema HW, Haesebrouck F, de Kruif A (2004). *In vitro* growth inhibition of major mastitis pathogens by *Staphylococcus chromogenes* originating from teat apices of dairy heifers. Vet Microbiol.

[CR26] Donelli G, Vuotto C, Mastromarino P (2013) Phenotyping and genotyping are both essential to identify and classify a probiotic microorganism. Microb Ecol Health Dis 24. 10.3402/mehd.v24i0.20105 10.3402/mehd.v24i0.20105 PMC3758930 24009545

[CR27] Dos Santos DC, Lange CC, Avellar-Costa P, Dos Santos KRN, Brito MAVP, Giambiagi-De Marval M (2016). *Staphylococcus chromogenes*, a coagulase-negative *staphylococcus s*pecies that can clot plasma. J Clin Microbiol.

[CR28] El-Jakee JK, Aref NE, Gomaa A, El-Hariri MD, Galal HM, Omar SA, Samir A (2013). Emerging of coagulase negative staphylococci as a cause of mastitis in dairy animals: an environmental hazard. Int J Vet Sci.

[CR29] FAO (2020) World Food and Agriculture - Statistical Yearbook 2020. In World Food and Agriculture - Statistical Yearbook 2020. FAO. 10.4060/cb1329en

[CR30] Fergestad ME, de Visscher A, L’Abee-Lund T, Chamba CN, Mainil JG, Thiry D, de Vliegher S, Wasteson Y (2021). Antimicrobial resistance and virulence characteristics in 3 collections of staphylococci from bovine milk samples. J Dairy Sci.

[CR31] Funari R, Shen AQ (2022). Detection and characterization of bacterial Biofilms and Biofilm-Based sensors. ACS Sens.

[CR32] Gurler H, Findik A, Sezener MG (2022). Determination of antibiotic resistance profiles and biofilm production of *Staphylococcus* spp. isolated from anatolian water buffalo milk with subclinical mastitis. Pol J Vet Sci.

[CR33] Heikkilä AM, Liski E, Pyörälä S, Taponen S (2018). Pathogen-specific production losses in bovine mastitis. J Dairy Sci.

[CR34] Hussein OH, Abdel-Hameed KG, El-Malt LM (2022). Prevalence and public health hazards of subclinical mastitis in dairy cows. Int J Vet Sci.

[CR35] Ibrahim ES, Dorgham SM, Mansour AS, Abdalhamed AM, Khalaf DD (2022). Genotypic characterization of *mecA* gene and antibiogram profile of coagulase-negative staphylococci in subclinical mastitic cows. Vet World.

[CR36] Isaac P, Bohl LP, Breser ML, Orellano MS, Conesa A, Ferrero MA, Porporatto C (2017). Commensal coagulase-negative *Staphylococcus f*rom the udder of healthy cows inhibits biofilm formation of mastitis-related pathogens. Vet Microbiol.

[CR37] Jacques M, Malouin F (2022). One health-one Biofilm. Vet Res.

[CR38] Jenkins SN, Okello E, Rossitto PV, Lehenbauer TW, Champagne J, Penedo MCT, Arruda AG, Godden S, Rapnicki P, Gorden PJ, Timms LL, Aly SS (2019). Molecular epidemiology of coagulase-negative *Staphylococcus* species isolated at different lactation stages from dairy cattle in the United States. PeerJ.

[CR39] Kirkan S, Göskoy E, Osman K (2005). Identification and Antimicrobial susceptibility of *Staphylococcus aureus* and Coagulase negative staphylococci from bovine Mastitis in the Aydin Region of Turkey. Turkish J Vet Anim Sci.

[CR40] Kizerwetter-Świda M, Chrobak-Chmiel D, Rzewuska M (2018). Current challenges of veterinary microbiological diagnostics concerning the susceptibility of staphylococci to antibiotics. Postepy Mikrobiologii.

[CR41] Koop G, de Visscher A, Collar CA, Bacon DAC, Maga EA, Murray JD, Supré K, de Vliegher S, Haesebrouck F, Rowe JD, Nielen M, Van Werven T (2012). Short communication: identification of coagulase-negative *Staphylococcus* species from goat milk with the API staph identification test and with transfer RNA-intergenic spacer PCR combined with capillary electrophoresis. J Dairy Sci.

[CR42] Kranjec C, Morales AD, Torrissen MM, Fernández L, García P, Kjos M, Diep DB (2021) Staphylococcal Biofilms: Challenges and Novel Therapeutic Perspectives. Antibiotics (Basel) 10:131. https://doi.org/.3390/antibiotics10020131 10.3390/antibiotics10020131 PMC7911828 33573022

[CR43] Lasagno M, Ortiz M, Vissio C, Yaciuk R, Bonetto C, Pellegrino M, Bogni C, Odierno L, Raspanti C (2018). Pathogenesis and inflammatory response in experimental caprine mastitis due to *Staphylococcus chromogenes*. Microb Pathog.

[CR44] Lee YJ, Lee YJ (2022). Characterization of biofilm producing coagulase-negative staphylococci isolated from bulk tank milk. Vet Sci.

[CR45] Lison J, Taratuta A, Paszenda Z, Szindler M, Basiaga M (2022). Perspectives in Prevention of Biofilm for Medical Applications. Coatings.

[CR46] Mahato S, Mistry HU, Chakraborty S, Sharma P, Saravanan R, Bhandari V (2017). Identification of variable traits among the methicillin resistant and sensitive coagulase negative staphylococci in milk samples from mastitic cows in India. Front Microbiol.

[CR47] Michels R, Last K, Becker SL, Papan C (2021). Update on coagulase-negative staphylococci—what the clinician should know. Microorganisms.

[CR48] Moraes GFQ, Cordeiro LV, de Andrade Júnior FP (2021). Main laboratory methods used for the isolation and identification of *Staphylococcus* spp. Rev Colon Cienc Quim Farm.

[CR49] Naqvi SA, de Buck J, Dufour S, Barkema HW (2018). Udder health in canadian dairy heifers during early lactation. J Dairy Sci.

[CR50] Nascimento JDS, Fagundes PC, Brito MAVDP, Netto Dos Santos KR, Bastos MDCDF (2005). Production of bacteriocins by coagulase-negative staphylococci involved in bovine mastitis. Vet Microbiol.

[CR51] Onni T, Vidili A, Bandino E, Marogna G, Schianchi S, Tola S (2012). Identification of coagulase-negative staphylococci isolated from caprine milk samples by PCR-RFLP of groEL gene. Small Rumin Res.

[CR52] Otto M (2004) Virulence factores of the coagulase-negative staphylococci. Front Biosci 1 9:841–63 10.2741/1295. 10.2741/1295 14766414

[CR53] Park JY, Fox LK, Seo KS, McGuire MA, Park YH, Rurangirwa FR, Sischo WM, Bohach GA (2011). Comparison of phenotypic and genotypic methods for the species identification of coagulase-negative staphylococcal isolates from bovine intramammary infections. Vet Microbiol.

[CR54] Pascu C, Herman V, Iancu I, Costinar L (2022) Etiology of mastitis and antimicrobial resistance in dairy cattle farms in the western part of Romania. Antibiotics 11(1). 10.3390/antibiotics11010057 10.3390/antibiotics11010057 PMC8772981 35052934

[CR55] Pedersen RR, Krömker V, Bjarnsholt T, Dahl-Pedersen K, Buhl R, Jørgensen E (2021). Biofilm Research in bovine Mastitis. Front Vet Sci.

[CR56] Persson WK, Aspán A, Nyman A, Persson Y, Grönlund AU (2011). CNS species and antimicrobial resistance in clinical and subclinical bovine mastitis. Vet Microbiol.

[CR57] Phe (2020) UK SMI ID 07: identification of *Staphylococcus* species, *Micrococcus* species and *Rothia* species

[CR58] Piccart K, Verbeke J, de Visscher A, Piepers S, Haesebrouck F, de Vliegher S (2016). Local host response following an intramammary challenge with *Staphylococcus fleurettii* and different strains of *Staphylococcus chromogenes* in dairy heifers. Vet Res.

[CR59] Piepers S, Opsomer G, Barkema HW, de Kruif A, de Vliegher S (2010). Heifers infected with coagulase-negative staphylococci in early lactation have fewer cases of clinical mastitis and higher milk production in their first lactation than noninfected heifers. J Dairy Sci.

[CR60] Piepers S, Schukken YH, Passchyn P, de Vliegher S (2013). The effect of intramammary infection with coagulase-negative staphylococci in early lactating heifers on milk yield throughout first lactation revisited. J Dairy Sci.

[CR61] Piessens V, de Vliegher S, Verbist B, Braem G, Van Nuffel A, de Vuyst L, Heyndrickx M, Van Coillie E (2012). Characterization of coagulase-negative *Staphylococcus* species from cows’ milk and environment based on *bap, icaA*, and *mecA* genes and phenotypic susceptibility to antimicrobials and teat dips. J Dairy Sci.

[CR62] Pinto RM, Lopes-De-Campos D, Martins MCL, van Dijck P, Nunes C, Reis S (2019). Impact of nanosystems in *Staphylococcus aureus* biofilms treatment. FEMS Microbiol.

[CR63] Pyörälä S, Taponen S (2009). Coagulase-negative staphylococci-emerging mastitis pathogens. Vet Microbiol.

[CR64] Raspanti CG, Bonetto CC, Vissio C, Pellegrino MS, Reinoso EB, Dieser SA, Bogni CI, Larriestra AJ, Odierno LM (2016). Prevalencia y sensibilidad a antibióticos de especies de estafilococos coagulasa negativos provenientes de mastitis subclínica en bovinos de tambos de la región central de Argentina. Rev Argent Microbiol.

[CR65] Rollin E, Dhuyvetter KC, Overton MW (2015). The cost of clinical mastitis in the first 30 days of lactation: an economic modeling tool. Prev Vet Med.

[CR66] Rosa NM, Penati M, Fusar-Poli S, Addis MF, Tola S (2022). Species identification by MALDI-TOF MS and gap PCR-RFLP of non-*aureus Staphylococcus, Mammaliicoccus*, and *Streptococcus* spp. associated with sheep and goat mastitis. Vet Res.

[CR67] Rudenko P, Sachivkina N, Vatnikov Y, Shabunin S, Engashev S, Kontsevaya S, Karamyan A, Bokov D, Kuznetsova O, Vasillieva E (2021). Role of microorganisms isolated from cows with mastitis in Moscow region in biofilm formation. Vet World.

[CR68] Ruiz-Ripa L, Gómez P, Alonso CA, Camacho MC, Ramiro Y, Puente J, Fernández-Fernández R, Quevedo M, Blanco JM, Báguena G, Zarazaga M, Höfle U, Torres C (2020). Frequency and characterization of antimicrobial resistance and virulence genes of coagulase-negative staphylococci from wild birds in Spain. Detection of tst-carrying *S. sciuri* isolates. Microorganisms.

[CR69] Ruiz-Romero RA, Martínez-Gómez D, Cervantes-Olivares RA, Díaz-Aparicio E, Ducoing-Watty AE (2020). Evaluation of pro- and anti-inflammatory interleukins in the mammary gland of goats experimentally infected with *Staphylococcus chromogenes*. Pol J Vet Sci.

[CR70] Sandholm M, Kaartinen L, Pyörälä S (1990). Bovine mastitis-why does antibiotic therapy not always work? An overview. J Vet Pharmacol Therap.

[CR71] Sawant AA, Gillespie BE, Oliver SP (2009). Antimicrobial susceptibility of coagulase-negative *Staphylococcus* species isolated from bovine milk. Vet Microbiol.

[CR72] Schukken YH, González RN, Tikofsky LL, Schulte HF, Santisteban CG, Welcome FL, Bennett GJ, Zurakowski MJ, Zadoks RN (2009). CNS mastitis: nothing to worry about?. Vet Microbiol.

[CR73] Silva V, Correia E, Pereira JE, González-Machado C, Capita R, Alonso-Calleja C, Igrejas G, Poeta P (2022). Exploring the Biofilm formation capacity in *S. pseudintermedius* and Coagulase-Negative Staphylococci Species. Pathogens.

[CR74] Simojoki H, Salomäki T, Taponen S, Livanainen A, Pyörälä S (2011). Innate immune response in experimentally induced bovine intramammary infection with *Staphylococcus simulans* and *S. epidermidis*. Vet Res.

[CR75] Souza FN, Piepers S, Della Libera AMMP, Heinemann MB, Cerqueira MMOP, De Vliegher S (2016). Interaction between bovine-associated coagulase-negative staphylococci species and strains and bovine mammary epithelial cells reflects differences in ecology and epidemiological behavior. J Dairy Sci.

[CR76] Souza RM, Souza FN, Batista CF, Piepers S, De Visscher A, Santos KR, Molinari PC, Ferronatto JA, Franca da Cunha A, Blagitz MG, Da Silva GG, Rennó FP, Cerqueira MMOP, Heinemann MB, De Vliegher D, Della Libera AMMP (2022). Distinct behavior of bovine-associated staphylococci species in their ability to resist phagocytosis and trigger respiratory burst activity by blood and milk polymorphonuclear leukocytes in dairy cows. J Dairy Sci.

[CR77] Srednik ME, Tremblay YDN, Labrie J, Archambault M, Jacques M, Cirelli AF, Gentilini ER (2017) Biofilm formation and antimicrobial resistance genes of coagulase-negative staphylococci isolated from cows with mastitis in Argentina. FEMS Microbiol Lett 364. 10.1093/femsle/fnx001 10.1093/femsle/fnx001 28087612

[CR78] Subramanian S, Huiszoon RC, Chu S, Bentley WE, Ghodssi R (2020). Microsystems for biofilm characterization and sensing – a review. Biofilm.

[CR79] Taher EM, Hemmatzadeh F, Aly SA, Elesswy HA, Petrovski KR (2020a) Survival of staphylococci and transmissibility of their antimicrobial resistance genes in milk after heat treatments. *LWT*, *129*. 10.1016/j.lwt.2020.109584

[CR80] Taher EM, Hemmatzadeh F, Aly SA, Elesswy HA, Petrovski KR (2020). Molecular characterization of antimicrobial resistance genes on farms and in commercial milk with emphasis on the effect of currently practiced heat treatments on viable but nonculturable formation. J Dairy Sci.

[CR81] Taponen S, Pyörälä S (2009). Coagulase-negative staphylococci as cause of bovine mastitis-not so different from *Staphylococcus aureus*?. Vet Microbiol.

[CR82] Toledo-Silva B, Beuckelaere L, De Visscher A, Geeroms C, Meyer E, Piepers S, Thiry D, Haesebrouck F, de Vliegher S (2022). Novel quantitative assay to describe *in vitro* bovine mastitis bacterial pathogen inhibition by non*-aureus* staphylococci. Pathogens.

[CR83] Tomazi T, Gonçalves JL, Barreiro JR, Arcari MA, dos Santos M (2015). Bovine subclinical intramammary infection caused by coagulase-negative staphylococci increases somatic cell count but has no effect on milk yield or composition. J Dairy Sci.

[CR84] Traversari J, van den Borne BHP, Dolder C, Thomann A, Perreten V, Bodmer M (2019) Non-*aureus* staphylococci species in the teat canal and milk in four commercial swiss dairy herds. Front Veterinary Sci 6(JUN). 10.3389/fvets.2019.00186 10.3389/fvets.2019.00186 PMC6582780 31249836

[CR85] Turchi B, Bertelloni F, Marzoli F, Cerri D, Tola S, Azara E, Longheu CM, Tassi R, Schiavo M, Cilia G, Fratini F (2020). Coagulase negative staphylococci from ovine milk: genotypic and phenotypic characterization of susceptibility to antibiotics, disinfectants and biofilm production. Small Rumin Res.

[CR86] Valckenier D, Piepers S, De Visscher A, De Vliegher S (2020). The effect of intramammary infection in early lactation with non-*aureus* staphylococci in general and *Staphylococcus chromogenes* specifically on quarter milk somatic cell count and quarter milk yield. J Dairy Sci.

[CR87] Valckenier D, Piepers S, Schukken YH, De Visscher A, Boyen F, Haesebrouck F, De Vliegher S (2021). Longitudinal study on the effects of intramammary infection with non-*aureus* staphylococci on udder health and milk production in dairy heifers. J Dairy Sci.

[CR88] Vanderhaeghen W, Piepers S, Leroy F, Van Coillie E, Haesebrouck F, de Vliegher S (2014). Invited review: Effect, persistence, and virulence of coagulase-negative Staphylococcus species associated with ruminant udder health. J Dairy Sci.

[CR89] Vanderhaeghen W, Piepers S, Leroy F, Van Coillie E, Haesebrouck F, De Vliegher S (2014). Invited review: Effect, persistence, and virulence of coagulase-negative *Staphylococcus* species associated with ruminant udder health. J Dairy Sci.

[CR90] Vanderhaeghen W, Piepers S, Leroy F, Van Coillie E, Haesebrouck F, de Vliegher S (2015). Identification, typing, ecology and epidemiology of coagulase negative staphylococci associated with ruminants. Vet J.

[CR91] Virdis S, Scarano C, Cossu F, Spanu V, Spanu C, de Santis EPL (2010) Antibiotic resistance in *Staphylococcus aureus* and coagulase negative staphylococci isolated from goats with subclinical mastitis. Vet Med Int 2010:517060. 10.4061/2010/517060 10.4061/2010/517060 PMC2860459 20445785

[CR92] Wei G, He Y (2022). Antibacterial and antibiofilm activities of novel cyclic peptides against methicillin-resistant *Staphylococcus aureus*. Int J Mol Sci.

[CR93] Windria S, Widianingrum DC, Salasia SIO (2016). Identification of *Staphylococcus aureus* and coagulase negative staphylococci isolates from mastitis milk of Etawa crossbred goat. Res J Microbiol.

[CR94] Wuytack A, De Visscher A, Piepers S, Boyen F, Haesebrouck F, De Vliegher S (2020). Distribution of non-*aureus* staphylococci from quarter milk, teat apices, and rectal feces of dairy cows, and their virulence potential. J Dairy Sci.

[CR95] Wuytack A, De Visscher A, Piepers S, Haesebrouck F, De Vliegher S (2020b) Fecal non-*aureus* staphylococci are a potential cause of bovine intramammary infection. Vet Res 51. 10.1186/s13567-020-00761-5 10.1186/s13567-020-00761-5 PMC7052973 32122405

[CR96] Zaatout N, Ayachi A, Kecha M, Kadlec K (2019). Identification of staphylococci causing mastitis in dairy cattle from Algeria and characterization of *Staphylococcus aureus*. J Appl Microbiol.

[CR97] Zigo F, Farkašová Z, Výrostková J, Regecová I, Ondrašovičová S, Vargová M, Sasáková N, Pecka-Kielb E, Bursová S, Kiss D (2022). Dairy cows’ udder pathogens and occurrence of virulence factors in Staphylococci. Animals.

